# Dynamics of Pollen Activation and the Role of H^+^-ATPase in Pollen Germination in Blue Spruce (*Picea pungens*)

**DOI:** 10.3390/plants9121760

**Published:** 2020-12-11

**Authors:** Maria Breygina, Ekaterina Klimenko, Alexandra Podolyan, Alexander Voronkov

**Affiliations:** 1 Department of Plant Physiology, Biological Faculty, Lomonosov Moscow State University, Leninskiye Gory 1-12, 119991 Moscow, Russia; kleo80@yandex.ru (E.K.); aleksaniaara@gmail.com (A.P.); 2 Timiryazev Institute of Plant Physiology, Russian Academy of Sciences, Botanicheskaya St. 35, 127276 Moscow, Russia; voronkov_as@mail.ru

**Keywords:** pollen germination, plant reproduction, H^+^-ATPase, pH, immunolocalization, gymnosperms, spruce

## Abstract

Pollen is a highly specialized structure for sexual plant reproduction. Early stages of pollen germination require the transition from dormant state to active metabolism. In particular, an important role during this early phase of angiosperm pollen germination is played by H^+^-ATPase. Very little is known about pollen activation in gymnosperm species, and information on the involvement of H^+^-ATPase is lacking. We tracked four indicators characterizing the physiological state of pollen: membrane potential, intracellular pH, anion efflux and oxygen uptake, in order to monitor the dynamics of activation in *Picea pungens*. Based on pH dynamics during activation, we assumed the important role of H^+^-ATPase in spruce pollen germination. Indeed, germination was severely suppressed by P-type ATPase inhibitor orthovanadate. In spruce pollen tubes, a pronounced pH gradient with a maximum in the apical zone was found, which was different from the pollen tubes of flowering plants. Using orthovanadate and fusicoccin, we found that the proton pump is largely responsible for maintaining the gradient. Immunolocalization of the enzyme in pollen tubes showed that the distribution of H^+^-ATPase generally coincides with the shape of the pH gradient: its maximum accumulation is observed in the apical zone.

## 1. Introduction

Pollen is a highly specialized haploid organism representing the crucial stage of the life cycle in seed plants; polar growth is the most studied feature of this life form. Another important feature of pollen is physiological dormancy which is typical for the majority of species, both angiosperms and gymnosperms [1]. Early stages of pollen morphogenesis require, thus, the transition of vegetative cells from dormant state to active metabolism. The next step is the formation of a pollen tube—a cell protrusion with a typical polarized structure: an apical domain with accumulated exocytic vesicles and a thin wall, a subapical area with an actin fringe, which keeps large organelles out of the apex, and a shank with mitochondria, ER and Golgi moving along the actin bundles, surrounded by a rigid wall [2]. Male gametophytes of angiosperms and conifers correspond in general terms to a similar structure plan but typical conifer pollen exhibits a wide range of features [3,4], among which are: a solid pollen wall, ability for bipolar germination [5] and different shape and size of the apical compartment, as well as the trajectory of organelle movement in pollen tubes [6,7,8,9], a rather sloping calcium gradient and membrane potential gradient [10,11], participation of microtubules in cyclosis [12] and specific arrangement of cell wall layers [13].

The transition of a vegetative cell from dormant state to active metabolism (called activation) has been studied mainly in angiosperms. In tobacco, lily, corn and *Arabidopsis* pollen, several events preceding the onset of polar growth have been described as follows: cytoplasm alkalinization and activation of respiration [14,15,16], efflux of K^+^ and Cl^−^ [17], influx of Ca^2+^ in the aperture area [18], plasma membrane hyperpolarization [19], starch synthesis [20], protein synthesis from mRNA accumulated before anthesis [21]. All these events take place during the first 10–30 min of germination in vitro.

Very little is known about pollen activation in most gymnosperm species. The rate of oxygen consumption was recorded for *Pinus mugo* [22], RNA and protein synthesis was found for *Pinus monticola* [23], early release of ROS was shown for *Picea pungens* [11] and intracellular ROS and NO were detected in *Cupressus arizonica* [24]. No information is yet available on other aspects of early germination. Early stages of pollen germination in conifers are difficult to study as (1) the pollen germinates for several hours or even days; and (2) pollen grains in many cases are covered with a continuous exine until one or several ruptures are formed [5], while most of the pollen grains in flowering plants have one or more apertures in the exine through which pollen tubes protrude [25].

An important role in the early germination phase of angiosperm pollen is played by H^+^-ATPase of the plasma membrane [15,26] which was shown by different approaches: intracellular pH measurement [14], inhibitory analysis [26], immunolocalization [27], patch-clamp [28], mutagenesis [29] and localization with GFP fusion [29,30]. This ATP-hydrolyzing enzyme translocates H^+^ from the cytoplasm to the apoplast, generating both electrical and [H^+^] gradients, thus energizing the plasma membrane [31].

Localization studies of tobacco, petunia and *Arabidopsis* show that the proton pump is present all along the tube length, except in the apical compartment, and petunia is most abundant in the subapical area [32], which corresponds well to the measured H^+^ fluxes [29,30] and intracellular pH gradient: the apical zone is acidic, followed by an alkaline band in the subapical area [33]. Mutants *aha6/8/9* lacking the enzyme exhibited premature pollen tube growth arrest, aberrant pollen tube phenotypes and compromised actin cytoskeleton organization at the tip [29]. Reversible inhibition of pollen tube growth with potassium cyanide (KCN) followed by the re-establishment of calcium, pH and secretion patterns [34] revealed the role of the pH gradient in pollen tube growth: the alkaline band was the first feature to re-establish, before secretion, the calcium gradient and growth. Acidification of lily pollen tubes with sodium acetate stopped apical growth and demonstrated interrelation between the alkaline band and the actin fringe: both structures moved into the apex as the acidic tip could no longer be detected [35]. Localization of two proteins associated with the actin fringe, ADF and AIP was also altered in acidified tubes [35]. Normally, ADF, perhaps dependent on AIP1, responds to alkaline pH, which is established in the subapical area, and stimulates local restructuring of the cytoskeleton [36,37]. Thus, pH can control the actin structure through actin-associated proteins and, therefore, maintain the proper secretion pattern. A study on *Arabidopsis* pollen tubes connected intracellular pH changes with anion transport over the pollen tube membrane [29,38]. A link between pH and calcium gradient also exists in angiosperm pollen tubes as dynamic actin microfilaments regulate Ca^2+^ channel activity and may consequently regulate cytoplasmic Ca^2+^ [39].

Pollen tubes of conifers can introduce us to a more ancient, low-rate polar growth type. No information is yet available on the pH gradient, H^+^-ATPase function and localization in the pollen of conifers.

## 2. Results

### 2.1. Dynamics of Pollen Activation

**Figure 1:**
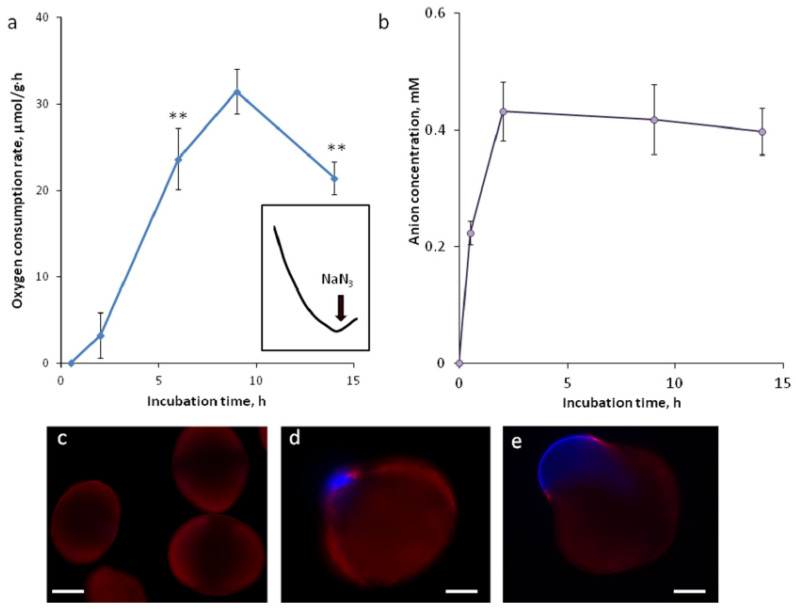
Dynamics of respiration and anion efflux during pollen activation in spruce. (**a**) Oxygen consumption rate measured by polarography in spruce pollen suspension (2 mg/mL), inset—typical respiration curve (9 h) after the addition of sodium azide; (**b**) anions released by pollen into the germination medium detected by MEQ fluorescence quenching; (**c**–**e**) typical pollen grains stained with wall-binding dyes Tinopal (intine, blue) and MitoSOX (exine, red) after 2 (**c**), 6 (**d**) and 9 (**e**) h of incubation. ** *p* < 0.01. Bar: 40 (**c**) and 20 µm (**d**,**e**).

We used several time points to monitor the activation process in spruce pollen in vitro. These points were chosen according to our previous study [5], in which we found that after 2 h, the pollen grain has a continuous exine (Figure 1c); at 6 h, the first exine ruptures appear (Figure 1d); after 9 h, small ruptures merge to obtain one or two large gaps through which the pollen tube initially comes out (Figure 1e); and at 14 h, the average pollen tube reaches, in length, the grain diameter. Nine hours is considered, thus representing the point of transition from activation to polar growth.

One of the most indicative signs of pollen activation is the increasing rate of oxygen consumption. We found that after 30 min of incubation, there is no detectable oxygen consumption; after 2 h, respiration is very low; and after 6 h, it is about seven times higher and reaches its peak at 9 h (Figure 1a). At 14 h, it is still high but significantly lower (*p* < 0.01). Sodium azide is added to an active suspension (9-h incubation) to show the correspondence between oxygen uptake and mitochondrial respiration (Figure 1a, inset).

Anion efflux, which is typical for angiosperm pollen, also takes place in *Picea*, and it occurs very quickly: after 30 min, the level of anions is significantly higher than before pollen incubation (*p* < 0.01); at 2 h, it is two times higher than at 30 min (*p* < 0.01), and no efflux is observed after 2 h, thus the curve reaches a plateau (Figure 1b).

### 2.2. Dynamics of Membrane Potential and Intracellular pH

**Figure 2:**
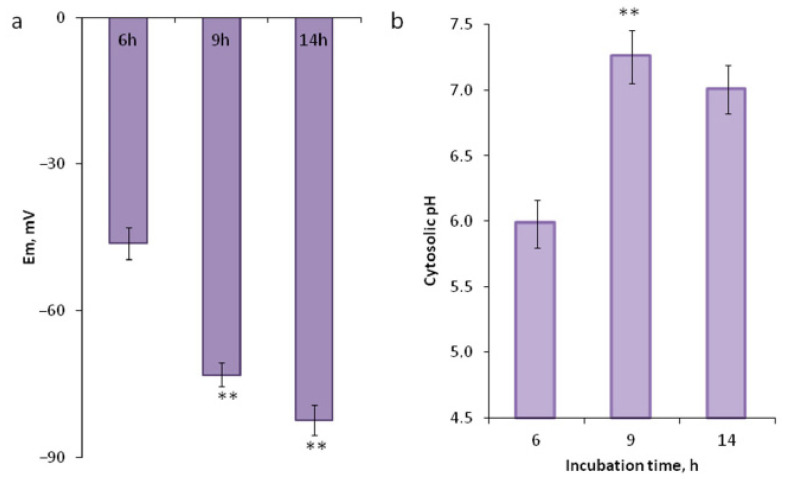
Dynamics of membrane potential and pH during pollen activation. (**a**) Plasma membrane hyperpolarization in the protrusion/tube assessed by DiBAC_4_(3) cytoplasmic staining. (**b**) Cytoplasm alkalization assessed by ratiometric BCECF-AM staining. Fluorescence was measured in the subapical area of the protrusion/tube. ** *p* < 0.01.

Membrane potential (MP) assessment revealed hyperpolarization during pollen germination. Due to the exine structure, we were not able to measure MP before 6 h as the MP-sensitive dye does not penetrate pollen grains with a continuous exine (Figure 1c) but during the interval of 6–14 h, we observed stable hyperpolarization with a slowdown after 9 h (Figure 2a). Intracellular pH was assessed at the same time points but showed different dynamics. After 6 h, when the ruptures appear, the cytoplasmic pH is still acidic (Figure 1b). After 9 and 14 h, the pH is stable and nearly neutral with a downward trend (Figure 2b).

**Figure 3:**
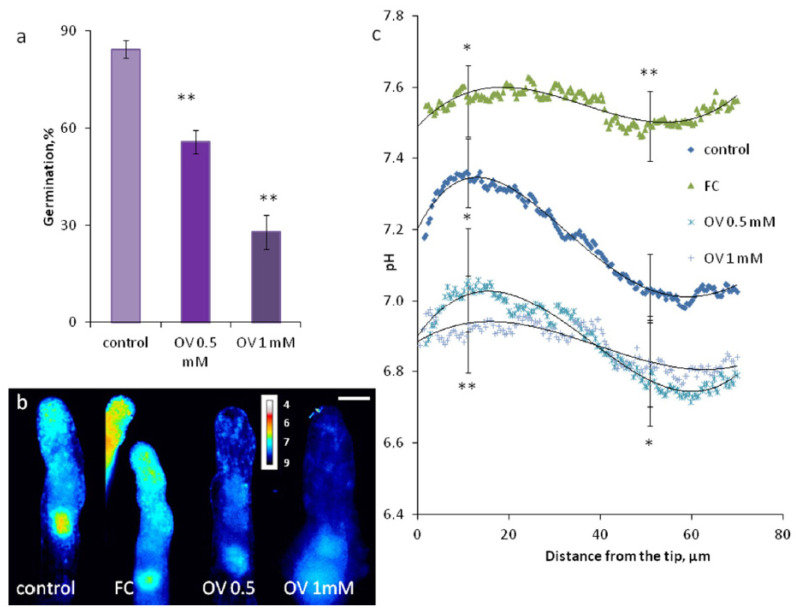
Intracellular pH in Picea pollen tubes. (**a**) Germination efficiency affected by inhibitor orthovanadate (OV); (**b**) pH distribution in typical pollen tubes treated by OV and H^+^-ATPase activator fusicoccin (FC); (**c**) pH distribution in pollen tubes treated with OV and FC, where each curve is an average from 21–53 pollen tubes. Statistics are shown for 2 points: 10 and 50 µm from the tip. ** *p* < 0.01; * *p* < 0.05. Bar: 20 µm.

Based on the observed dynamics of pH and MP, we can assume that H^+^-ATPase activation occurs between 6 and 9 h of incubation. An important role of this enzyme was found in experiments with its inhibitor sodium orthovanadate (OV) (Figure 3a): while in the control, suspension germination efficiency was close to 90% (20-h incubation), 500 µM OV significantly inhibited pollen germination (<60%) and 1 mM OV reduced germination to less than 30%.

### 2.3. pH Gradient in Growing Pollen Tubes

We used pH-sensitive ratiometric BCECF staining to assess the intracellular pH distribution in pollen tubes, which reveals possible effects of the H^+^-ATPase inhibitor and activator on this profile. We found a pH gradient with one maximum close to the tip area (7.36 ± 0.10) and a neutral plateau along the tube shank (7.03 ± 0.11) (Figure 3b,c). The participation of the proton pump in maintaining the gradient was shown using OV and H^+^-ATPase activator fusicoccin (FC): both influences altered the shape of the gradient. Vanadate induced acidification of the pollen tube cytoplasm, which was significant in the apical area (10 µm from the tip); two concentrations (500 µM and 1 mM) had a similar effect. Fusicoccin caused the opposite effect, namely, alkalization, which was more pronounced in the distal part of the tube (50 µm from the tip). Both actions led to the equalization of pH along the tube, that is, to the dissipation of the gradient. Thus, maintenance of the gradient is largely the result of H^+^-ATPase activity.

### 2.4. Immunolocalization of H^+^-ATPase in Pollen Tubes

**Figure 4:**
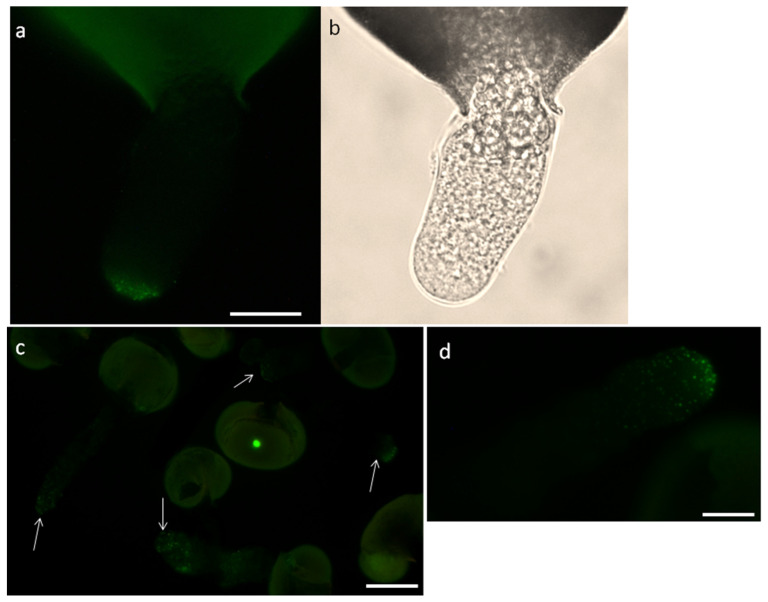
Immunolocalization of H^+^-ATPase in *Picea* pollen tubes. (**a**) Short pollen tube with the antibody signal localized in the apical zone (pointed by arrows); (**b**) the same tube in brightfield; (**c**) suspension with longer pollen tubes; (**d**) typical pollen tube with maximum fluorescent signal located close to the tube tip. Bar: 20 µm.

Immunolocalization of H^+^-ATPase showed its predominantly apical localization in short and longer pollen tubes (Figure 4). This pattern was in good alignment with the pH profile in pollen tubes of the same length (Figure 3b,c): the alkaline part of the tube corresponds to the maximum density of the enzyme on its surface, except for the first 3 μm, where, presumably, the activity of H^+^-ATPase is suppressed by high calcium. In FC-treated tubes, this zone is absent.

**Figure 5:**
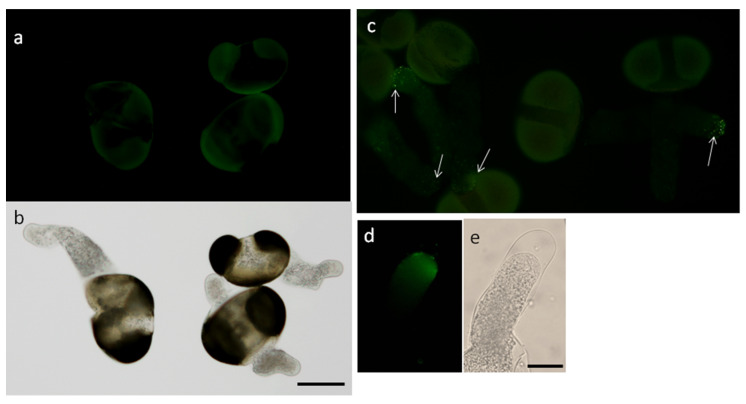
Testing antibodies for specificity. (**a**) Suspension treated with pre-immune rabbit serum + goat anti-rabbit AlexaFluor secondary antibodies—no signal in pollen tubes is detected, only weak exine autofluorescence; (**b**) the same pollen in brightfield; (**c**) suspension treated with rabbit anti- H^+^-ATPase primary antibodies + goat anti-rabbit AlexaFluor secondary antibodies. Fluorescent signal can be seen almost in all pollen tubes (arrows); (**d**,**e**) plasmolized pollen tube showing signal localization in the tube apex (focus on the cell wall). Bar: 50 (**a**–**c**) and 20 µm (**d**,**e**).

Experiments with pre-serum were made to control the specificity of primary antibodies: pollen tubes from the same suspension were treated simultaneously with pre-serum (Figure 5a,b) and anti- H^+^-ATPase antibodies followed, in both cases, with secondary antibodies. In the first case, no fluorescence in pollen tubes is detected; in the second case, we see rather intensive staining almost in all pollen tubes (Figure 5c). Experiments with plasmolized pollen tubes showed the absence of staining in the apoplast/cell wall (Figure 5d,e, focus is on the cell wall) and, presumably, staining of the plasma membrane and H^+^-ATPase-containing exocytic vesicles, but additional experiments to refine localization using alternative methods would be desirable in future studies.

## 3. Discussion

One of the key differences between pollen tube growth in gymnosperms and angiosperms is the germination rate which depends on the certain species studied but, in general, is much slower in conifers [7]. For example, of the two species we cultivated in vitro, spruce pollen needs about 20 times more time to germinate than tobacco pollen (9 h vs. 30 min). We chose several time points according to previously studied morphological changes in *Picea* pollen so that they would allow us to compare the activation process with that in model angiosperm species. The comparison described in the text is summarized in Table 1.

The dynamics of oxygen consumption in *Picea pungens* is different from that reported previously for tobacco [15]: instead of a sharp rise in the respiration level with saturation, we observe a curve with a maximum at the tube exit point (9 h). The dynamics of anion release was quite similar to that reported for tobacco [40] considering the differences in the germination rate: in both species, the efflux was finished before the activation actually began, ahead of other physiological changes detected. Due to the slower activation in spruce, we managed to fix a point with an intermediate level of anions (30 min—Figure 1b), while the process of tobacco went too fast and was completed after 2 min of incubation [19]. Membrane potential (MP) assessment revealed hyperpolarization during pollen germination in spruce, as was earlier described for tobacco and lily [41] with comparable meanings, though in spruce, the difference in values was greater, at least at the activation stage (from −46 to −73 in spruce vs. −37 to −45 in tobacco). One possible explanation is that a certain range of MP values is a species-specific parameter depending, for example, on pollen size (spruce pollen is large); the second is that the correspondence of stages is not complete and we cannot compare them directly. Regardless of this, observing hyperpolarization in all studied species, we can conclude that this process is universal among seed plants. Comparing the absolute values of the membrane potential measured in pollen grains of various species [27,42,43,44,45], we can note that the values obtained for spruce lie within the range observed in flowering plants.

Comparing the dynamics of intracellular pH and oxygen uptake, we see an obvious similarity. This fact is a good example of active ATP expenditure for the operation of the proton and calcium ATPases in pollen, along with other energy-consuming processes such as endocytosis, exocytosis and actin cytoskeleton dynamics [46]. The dynamics of intracellular pH was quite similar in tobacco and spruce: alkalinization of the cytoplasm was observed during the activation process, reaching a plateau near the moment of tube emergence (20 min in tobacco vs. 9 h in spruce) [15]. In tobacco, it was shown that H^+^ efflux from the pollen grain as well as in the pollen tube (region of the alkaline band) is mainly caused by vanadate-sensitive H^+^-ATPase [30]. H^+^-ATPase is thought to be the key enzyme driving pH shifts and plasma membrane hyperpolarization in angiosperm pollen, therefore providing an ionic basis for metabolism activation [28,47,48]. In different angiosperm species, antagonists of plasma membrane H^+^-ATPase inhibited pollen germination and tube growth, whereas the stimulator of the enzyme was able to boost the germination frequency as well as the tube growth rates [30,49]. Stimulation of germination by 20% was achieved with fusicoccin, which promotes binding of 14-3-3 proteins to P-type H^+^-ATPase [48]. Here, we report the role of H^+^-ATPase in the activation of spruce pollen: OV severely inhibited pollen germination. Thus, we can conclude that the activation mechanism is universal for seed plants from different taxonomic groups.

**Table 1:** Spatial and dynamical changes in physiological parameters in angiosperms and spruce.

Physiological Indicators	Tobacco/Lily/*Arabidopsis*/Petunia	Spruce
Oxygen consumption dynamics	Sharp rise in respiration level with saturation before the tube exit point [15]	Curve with a maximum at the tube exit point
Anion release dynamics	Early efflux before the activation [19]	
Membrane potential dynamics	Hyperpolarization during activation and tube emergence [41]	
Intracellular pH dynamics	Alkalinization during the activation, reaching a plateau at the tube exit point [15]	
pH gradient in the pollen tube	Maximum in subapical area; minimum at the apex [33,50]	Maximum in the apical area
H^+^-ATPase localization	Absent in the apical membrane; present along the shank [29,30,32]	Located predominantly in the apical area

In pollen tubes of flowering plants, important functions of plasma membrane H^+^-ATPase include maintaining the pH gradient in the cytoplasm and MP gradient on the plasmalemma as well as osmoregulation [26,29,30]. The gradient in these tubes has a rather complex shape: at the tip, the pH is acidic; in the subapical area, there is an alkaline band; and along the rest of the tube, the pH is quite stable and tends to neutral values [33,50]. In spruce, the shape of the pH gradient is different: the pH maximum is adjacent to the apical area and in the tube shank, pH is neutral. This difference is in accordance with observed localization of H^+^-ATPase in *Picea*: the highest density of the protein was observed in the apical zone, while in Arabidopsis and tobacco, the protein was excluded from the tip [29,30], and in petunia, maximum accumulation was reported in the subapical area [32]. The close relationship between the proton pump and the pH gradient was confirmed by inhibitory analysis: both OV and FC altered the shape of the gradient. Vanadate induced acidification of the cytoplasm of the tube, which was significant in the apical area (where the protein is the most abundant) and the equalization of pH along the tube. FC caused alkalization and dissipated the gradient as well. The observed pattern corresponds well with the fact that the spruce pollen tube possesses an OV-sensitive membrane potential gradient with the maximum adjacent to the apical plasma membrane [11].

Thus, maintenance in spruce pollen tubes is largely the result of the proton pump activity, but the pattern of H^+^-ATPase distribution differs drastically from that in angiosperm pollen tubes. The discovered pattern of the pH gradient fits well into a number of features of coniferous pollen tubes related to cytoplasm zoning. These are the important differences between the pollen tubes of conifers and flowering plants, including: the apical zone enriched in mitochondria and endomembrane system components instead of an inverted vesicle cone [7], a two-fold tip-focused [Ca^2+^] gradient [10], the direct fountain pattern of organelle movement [9] and the presence of cellulose and callose in the tip of the tube [13]. It can be assumed that the phenomenon of a segregated “angiosterm-type” apical compartment filled with vesicles, exhibiting a steep [Ca^2+^] gradient, separated from the rest of the cytoplasm by the actin fringe and covered with a pectin wall is associated with the absence of H^+^-ATPase in the apical membrane and resulting in an acidic tip and subapical alkaline band. We can suggest that the alternative pattern of the H^+^-ATPase distribution of the proton pump and cytoplasmic pH is one of the factors that cause faster tube growth in flowering plants as compared to conifers. The relationship between these factors remains to be studied.

## 4. Materials and Methods

### 4.1. Plant Material 

Male cones of *Picea pungens* Engelm. were collected from several trees in Moscow State University Botanical Garden (May–June 2020) and kept at room temperature until the scales opened to shed pollen, which was then stored at −20 °C.

### 4.2. Pollen Germination Efficiency

Pollen germination efficiency was assessed after 20 h of incubation at 25 °C in standard medium: 0.3 M sucrose, 1 mM H_3_BO_3_ and 1 mM CaCl_2_ in 15 mM MES-Tris buffer (pH 5.5) [8] on a mini-rocker shaker MR-1 (Biosan, Moscow, Russia) at low speed. Pollen grains were fixed with 2% paraformaldehyde in 50 mM K-phosphate buffer (pH 7.4).

### 4.3. Immunolocalization 

Immunolocalization was performed according to a previously reported protocol [32]. Primary antibodies: rabbit anti-H^+^-ATPase antibodies (Agrisera, AS07 260; 1:300 dilution in 10 mM PBS + 1% BSA); secondary antibodies: goat anti-rabbit lgG (H + L), Alexa Fluor Plus 488 conjugated antibodies (Invitrogen; 1:300 dilution in 10 mM PBS + 1% BSA). Specificity was checked with rabbit pre-immune serum (Agrisera, AS07 260PRE). All of the experiment was performed as described earlier including staining with secondary antibodies.

### 4.4. Respiration Assessment 

Oxygen uptake was measured polarographically with Clark-type oxygen electrode and liquid analyzer Expert 001 (Econix-Expert Ltd., Moscow, Russia). Pollen samples (16 mg) in SM were incubated in the measurement cell (V = 3.2 mL).

### 4.5. Anion Release 

Anion release from pollen grains to solution was studied using fluorescent dye 6-methoxy-N-ethylquinolinium iodide (MEQ; Molecular Probes, Carlsbad, CA, USA). Pollen grains were removed by filtration (nylon filter 11 µm) and MEQ was added to the incubation medium to a final concentration of 5 µM. Staining was performed for 20 min in the dark at 25 °C. The dye fluorescence was measured on an RF-5301PC spectrofluorometer (Shimadzu, Kioto, Japan). Fluorescence was excited at 344 nm and recorded at 445 nm. The autofluorescence of the incubation medium was negligible. Calibration was performed in SM with standard KCl concentrations.

### 4.6. Membrane Potential Assessment 

Absolute MP values were determined according to a previously reported protocol [51]. Cells were stained with DiBAC_4_(3) (Bis-(1,3-dibutylbarbituric acid) trimethine oxonol, molecular probes) (5 µM) for 10 min. Since the dye must stain the cytoplasm, cells with a solid exine were not suitable for measurements as the dye could not be loaded properly.

### 4.7. Assay of Cytoplasmic pH 

For pH assessment, pollen tubes were stained with 10 μM BCECF-AM (Thermo Fisher Scientific, San Jose, CA, USA) at 25 °C for 10 min as previously reported for *Lilium longiflorum* [50]. Fluorescence (515–565 nm) was excited in two channels: I_1_ = 475 − 495 nm; I_2_ = 426 − 446 nm. I_1_/I_2_ is proportional to the pH value. Calibration was performed according to [33]. Since the dye must stain the cytoplasm, cells with a solid exine were not suitable for measurements as the dye could not be loaded properly.

### 4.8. Fluorescent Pollen Wall Staining

Intine was stained with 10 µM Calcofluor white M2R (Sigma, Darmstadt, Germany). Exine sporopollenin was visualized with 5 µM MitoSOX (Thermo Fisher Scientific, San Jose, CA, USA) as reported previously [52].

### 4.9. Microscopy and Image Analysis 

For quantitative fluorescence microscopy, an Axioplan 2 imaging MOT (Zeiss, Oberkochen, Germany) wide-field microscope, AxioCam HRc camera and a high-speed shutter were operated with AxioVision 4.7.10; images were processed with the same software. Qualitative fluorescence and brightfield images were obtained with an ADF Pro08 digital camera (ADF optics, Hangzhou, China) on the same microscope.

### 4.10. Statistical Analysis 

Average meanings and standard errors are presented in figures. Significance was evaluated by Student’s *t* test (** *p* < 0.01; * *p* < 0.05) and is shown on diagrams.
